# A case of a painful coalition between pisiform and hamate

**DOI:** 10.1080/23320885.2019.1576132

**Published:** 2019-02-21

**Authors:** Atsuyuki Inui, Yutaka Mifune, Hanako Nishimoto, Takahiro Niikura, Ryosuke Kuroda

**Affiliations:** Department of Orthopedic Surgery, Kobe University Graduate School of Medicine, Kobe, Japan

**Keywords:** carpal coalition, pisiform, hamate

## Abstract

We report a case of the pisiform and hamate coalition in 13-years-old male. Interoperative findings revealed that the ulnar nerve runs over the hook of the hamate which is the nonanatomic site. Surgical resection of pisiform relieved the pain.

## Introduction

Fusions of the carpal bones are rare anomalies which occur in 1% of the population [1]. Carpal coalition is usually an asymptomatic entity diagnosed by a hand radiograph by chance. Coalitions may occur anywhere in the carpus, but the most common variant occurs between the lunate and triquetral bones followed by the capitate and hamate [2]. Fusion between the pisiform and hamate bones was first described by Cockshott [3]. To date, about ten cases of pisiform and hamate coalition have been reported in English literature. Here we report a case of a painful coalition between the pisiform and hook of the hamate in a teenaged patient.

## Case

**Figure 1.**
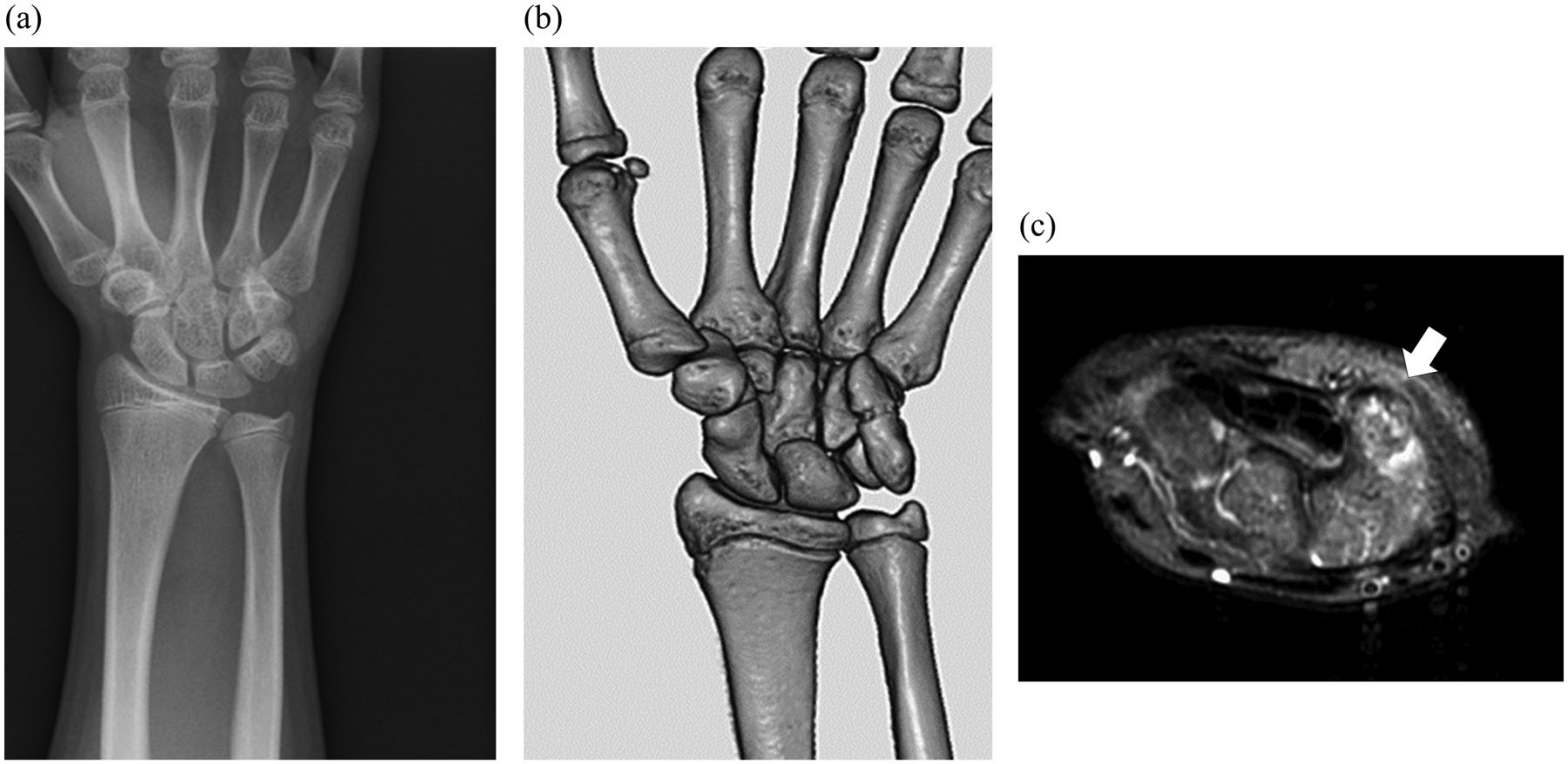
(a) Plane X-ray before the surgery (b) 3D reconstruction CT scan before the surgery (c) T2 image of MRI; white arrow indicates pisiform.

**Figure 2.**
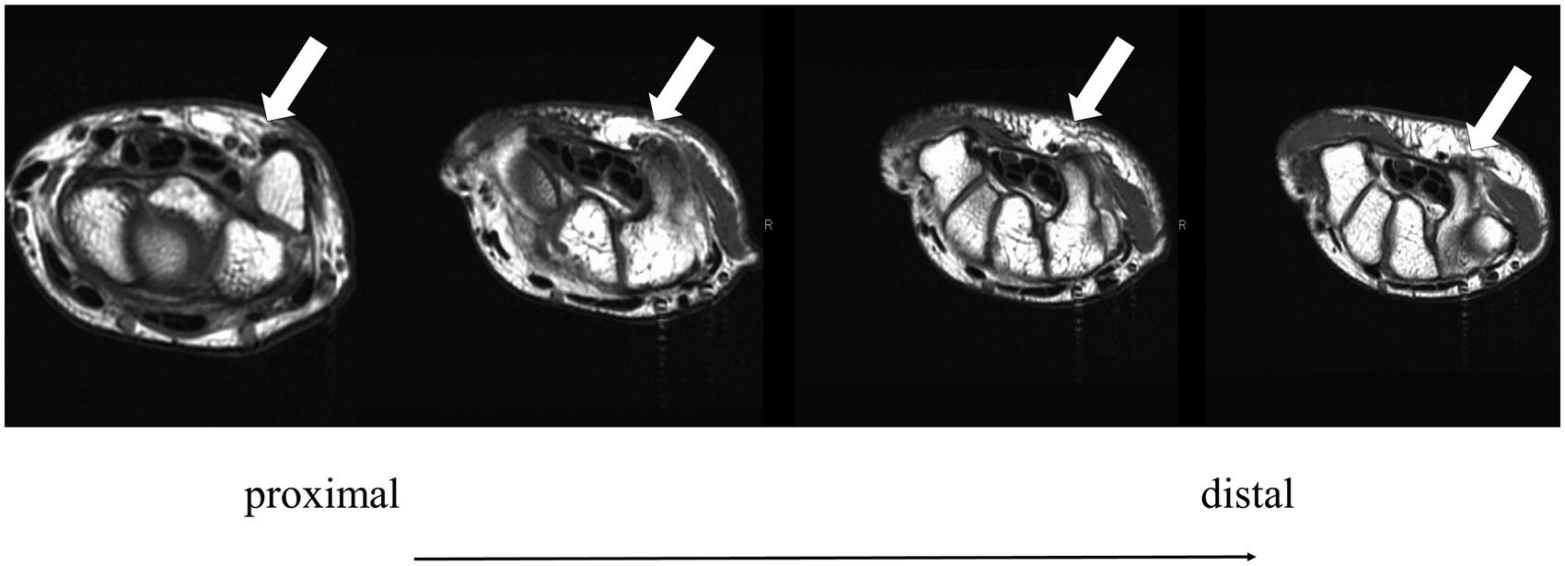
MRI findings revealed that the ulnar nerve (white arrow) run over the hook of hamate.

**Figure 3.**
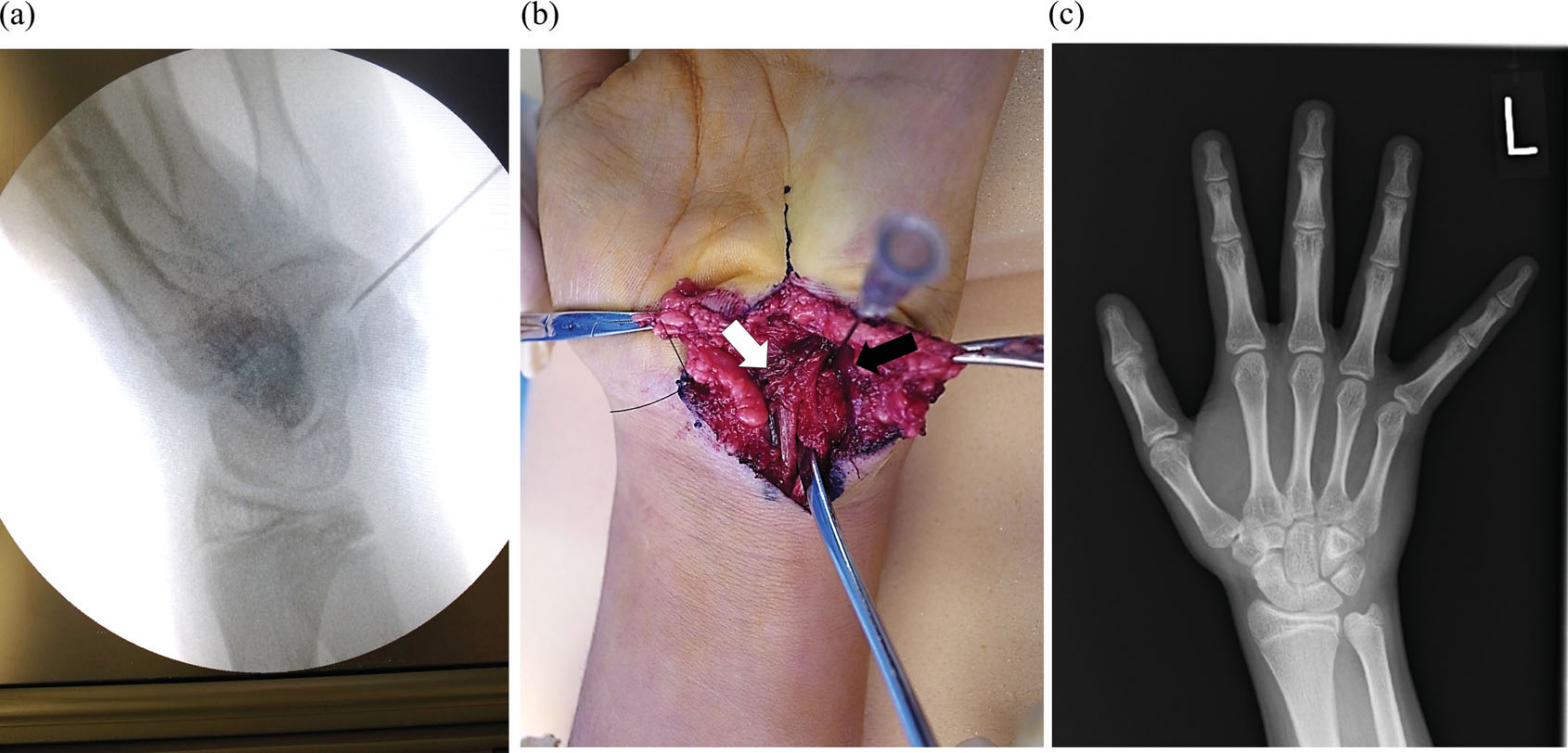
(a) X-ray imaging during the surgery; the needle was inserted between pisiform and hamate (b) Interoperative view; ulnar nerve (white arrow) is retracted radial to the coalition (black arrow). The nerve runs over the hook of the hamate. (c) Plane X-ray two years after the surgery.

A 13-year-old boy was referred to the clinic because of the left wrist joint pain without apparent trauma. Physical examination revealed tenderness over the pisiform, and the pain worsened when the patient flexed the wrist joint. He had neither the limited range of motion of wrist nor the unstable distal radioulnar joint. There were no signs of ulnar nerve neuropathy. The radiography and computed tomography (CT) of the wrist joint showed an unusual shape of the pisiform as well as an incomplete coalition between the pisiform and hamate (Figure 1(a,b)). Injection of local anaesthetic with steroids relieved the pain for several months. However, 8 months later, he showed up to the clinic again because of pain recurrence. By CT, the progression of fusion between the pisiform and hamate was observed. Magnetic resonance imaging (MRI) showed bone oedema around the pisiform in T2 fat suppression imaging (Figure 1(c)). The axial view of the wrist joint revealed that the patient’s ulnar nerve runs over the hook of the hamate, which should run between the pisiform and hook of the hamate (Figure 2). The injection of an analgesic did not relieve his pain; thus, surgical resection of the pisiform was performed. Prior to the skin incision, the position of the ulnar nerve was marked on the skin by ultrasound. Under general anaesthesia, the ulnar nerve was taped proximal to the coalition site. The nerve, which runs over the hook of the hamate to the radial side was then carefully retracted. There was a fibrous connection between the pisiform and hamate (Figure3(a,b)), and the pisiform was removed in one block. After skin closure, the short palmer splint was adopted for 2 weeks. He realised muscle weakness of finger abduction without any sensory disturbance, which spontaneously healed over several weeks. Two years after surgery, he had no pain, and the range of motion of the wrist joint was normal. The follow-up radiograph showed normal alignment of the remaining carpal bones (Figure3(c)).

## Discussion

The geographical variation of carpal bone coalitions has been reported, indicating that genetic factors may play a role in coalition [4]. The absence of joint cavitation during the embryological period and chondrification of the joint interzone is thought to lead to this congenital coalition. However, this mechanism does not explain the pisiform–hamate coalition. As a sesamoid, the pisiform forms no cartilaginous connection to the hamate throughout its entire period of ossification [5]. This rare anomaly is considered to result from the metaplastic conversion of ligament into bone [6]. As first devised by Minaar in 1952 [7] to categorise the lunate-triquetrum fusions based on radiographs, Minaar’s classification has been used to categorise all carpal coalitions. Type 1 is characterised by incomplete fusion resembling pseudarthrosis. Type 2 is characterised by fusion with a notch of varying depths at the site of the usual division between the two bones. Type 3 is characterised by complete fusion, and with type 4, there is complete fusion associated with other carpal anomalies. Most of the reported symptomatic cases were type 1 or 2 coalition because minor movement between the pisiform and hamate can cause cartilage degeneration and pain. In contrast, most asymptomatic pisiform–hamate coalitions previously reported were types 3 and 4 [6].

Excision of the pisiform has been reported to treat pisotriquetral complaints [8]. Berkowitz *et al.* also demonstrated two cases of the pisiform–hamate fusion which caused ulnar neuropathy. Excision of the pisiform, combined with the decompression of the Guyon canal has been previously reported [9]. On the other hand, Zeplin et al. reported screw fixation between the pisiform and hamate to achieve complete fusion [2]. In the present case, we observed type 1 coalition and speculated that the minor movement between pisiform and hamate caused pain. Because we were unsure whether bone fusion could be achieved with screw fixation, excision of pisiform was performed. Preoperative MRI and interoperative findings revealed that the ulnar nerve ran over the hook of the hamate, which is a nonanatomic site. The surgeon must be aware that this type of coalition can cause ulnar wrist pain. Moreover, the ulnar nerve must be taken care of during surgical treatment.
